# Assessing Specific Cognitive Deficits Associated with Dementia in Older Adults with Down Syndrome: Use and Validity of the Arizona Cognitive Test Battery (ACTB)

**DOI:** 10.1371/journal.pone.0153917

**Published:** 2016-05-12

**Authors:** Amanda Sinai, Angela Hassiotis, Khadija Rantell, Andre Strydom

**Affiliations:** 1 Division of Psychiatry, University College London, London, England; 2 Camden Learning Disability Service, Camden and Islington NHS Foundation Trust, London, England; 3 Institute of Neurology, Education Unit, Queens Square, University College London, London, England; 4 Islington Learning Disability Partnership, Camden and Islington NHS Foundation Trust, London, England; 5 The London Down Syndrome Consortium (LonDownS), London, England; Maastricht University, NETHERLANDS

## Abstract

**Background:**

Down syndrome is associated with specific cognitive deficits. Alongside this, older adults with Down syndrome are a high risk group for dementia. The Arizona Cognitive Test Battery (ACTB), a cognitive assessment battery specifically developed for use with individuals with Down syndrome, has been proposed for use as outcome measures for clinical trials in this population. It has not been validated in older adults with Down syndrome. This study aims to assess the use and validity of the ACTB in older adults with Down syndrome.

**Methods:**

Participants with Down syndrome aged 45 and over were assessed using the ACTB, standard tabletop tests and informant ratings.

**Results:**

Assessment outcomes of 49 participants were analysed. Of these, 19 (39%) had a diagnosis of dementia or possible dementia. Most participants were able to attempt most of the tasks, although some tasks had high floor effects (including CANTAB Intra-Extra Dimensional shift stages completed and Modified Dots Task). Of the ACTB tasks, statistically significant differences were observed between the dementia and no dementia groups on CANTAB Simple Reaction Time median latency, NEPSY Visuomotor Precision—Car and Motorbike and CANTAB Paired Associates Learning stages completed. No significant differences were observed for CANTAB Intra-Extra Dimensional Shift, Modified Dots Task, Finger Sequencing, NEPSY Visuomotor precision—Train and Car and CANTAB Paired Associates Learning first trial memory score. Several of the tasks in the ACTB can be used in older adults with Down syndrome and have mild to moderate concurrent validity when compared to tabletop tests and informant ratings, although this varies on a test by test basis.

**Conclusions:**

Overall, scores for a number of tests in the ACTB were similar when comparing dementia and no dementia groups of older adults with Down syndrome, suggesting that it would not be an appropriate outcome measure of cognitive function for clinical trials of dementia treatments without further modification and validation.

## Introduction

Down syndrome is associated with a number of characteristic features, including characteristic facial features and specific physical health problems, such as cardiac defects, thyroid problems and gastrointestinal and immunological disorders. It has been estimated to have a rate of approximately 14 per 10,000 live births in the USA [1] and is the most common genetic cause of intellectual disability.

Down syndrome is also associated with a specific cognitive profile [2] which includes impairments in the prefrontal and hippocampal domains [3], such as a relative weakness in verbal short term memory [4], with disproportionally smaller brain volume in these areas [5]. People with Down syndrome also have smaller cerebellar volumes [6], although the research on cerebellar cognitive function in humans is limited.

In younger adulthood, it may be that IQ (Intelligence Quotient) in people with Down syndrome remains fairly stable [7]. However, it has become more evident that one of the behavioural phenotypes of older adults with Down syndrome is increased risk of developing dementia [8]. This is often a dementia of Alzheimer's type (DAT). At 65 years of age, the risk for people with Down syndrome developing dementia has been estimated to be 80% [9], although there is large variation between individuals—some develop signs and symptoms of Alzheimer’s disease in their 40s, but there have also been reports of individuals without dementia at age 70 [10].

Dementia is characterised by a decline from baseline functioning in memory and other cognitive and daily living skills. As in the general population, decline in cognitive skills are important symptoms when making a diagnosis of dementia in people with Down syndrome, although there are some differences in the clinical presentation in this population, for example the high rates of epilepsy seen in those with Down syndrome and dementia [9].

### Cognition in older adults with Down syndrome

There are a number of studies that have investigated cognition in older adults with Down syndrome with and without dementia. A study by Ball and colleagues which included 25 participants with Down syndrome and dementia of Alzheimer’s type (DAT) and 78 participants with Down syndrome without dementia showed that participants with dementia had impaired performance on measures of executive functioning and memory compared to those without dementia [11]. Cognitive functions have been shown to decline sequentially in people with Down syndrome, with different cognitive functions being affected at different stages of the dementia [12]. A longitudinal study in the USA showed severely diminished verbal long term storage and retrieval processing abilities in those with early stage dementia compared to those without a diagnosis of dementia [13].

Older adults with Down syndrome have also been shown to perform worse on cognitive tests, particularly those requiring planning and attention, when compared to younger adults with Down syndrome and both older and younger adults with non-Down syndrome intellectual disability [14].

In those with cognitive deterioration, decreases in executive function and significant changes in behaviour have been shown to occur, which are not just due to the memory decline [15]. Although forgetfulness and confusion are common early symptoms, general slowness and frontal lobe related features, including diminished initiative and social withdrawal also present early in dementia in people with Down syndrome [16], to the extent that adults with Down syndrome may meet the criteria for a dementia of frontal type before they progress to meeting the criteria for Alzheimer’s disease [17].

A recent study has identified development of cerebellar motor signs, in particular an ataxic syndrome, during the progression of dementia and epilepsy in this population [18].

These studies suggest that cognitive tests focused on cognitive abilities associated with prefrontal and hippocampal brain areas should help to identify the early stages of dementia in older people with Down syndrome.

### Assessment of cognition

Advances in pharmacological treatments for Alzheimer’s disease and the recent trial of memantine in older adults with Down syndrome [19] have highlighted the need to obtain acceptable, validated and standardised measures of cognitive function in people with Down syndrome. These measures can help clarify a diagnosis of dementia, and are also required to measure outcomes, both clinically and in research, as the emphasis shifts towards prevention and treatment. Despite a large variety of cognitive assessment tools for diagnosis of DAT in people with Down syndrome, there is currently no consensus on how best to assess and track decline associated with dementia in this population [20].

The Arizona Cognitive Test Battery (ACTB) was developed to assess cognitive function in people with Down syndrome, focusing on cognitive difficulties associated with the prefrontal, hippocampal and cerebellar areas [21]. It has been validated for use in both laboratory and home environments and makes use of non-verbal responses, in order to reduce confounding by poor verbal ability. It is largely based on use of a touchscreen computer interface, and incorporates several well-known CANTAB (Cambridge Neuropsychological Test Automated Battery) cognitive tests. In an attempt to overcome potentially high floor levels in this population, several of the tasks include measurements of errors as well as measures of success.

The ACTB is becoming increasingly recognised as a useful cognitive assessment battery for people with Down syndrome [22, 23]. It has been validated in children and adults with Down syndrome, up until the age of 38. However, it has not been validated in older adults with Down syndrome, and it is not known if the battery would be of value in assessing for dementia and whether it can detect differences between older adults with and without dementia.

It is also proposed that the ACTB could be a useful measure of outcomes in treatment trials in this population, for example of dementia treatments, but the psychometric properties and floor effects of the ACTB need to be confirmed in older adults.

### Aim and Hypothesis

This study aimed to assess the use and validity of the ACTB in older adults with Down syndrome (with and without clinical diagnoses of dementia), compared to traditional tabletop tests and informant ratings.

Our Primary Hypothesis was that the ACTB is able to detect differences in cognitive functions involving the prefrontal, hippocampal and cerebellar regions between people with Down syndrome who have dementia and those who do not have dementia.

## Materials and Methods

The study was approved by the National Research Ethics Committee and conducted in accordance with the Mental Capacity Act [24]. NHS (National Health Service) Research and Development and local NHS site permissions were also granted.

Capacity to consent was assessed according to the Mental Capacity Act [24]. The Mental Capacity Act has clear specifications about research with participants who may lack capacity. This includes identifying a consultee in cases where participants lack capacity to consent. Participants (or their consultee if the participant lacked capacity) provided written consent. This consent procedure was approved by the NHS Research Ethics Committee.

### Setting

The study was conducted in the Greater London area and surrounding counties. This is an area with a diverse population, including a large range of ethnic and socio-economic backgrounds.

### Recruitment

Professionals working in community intellectual disability teams, a specialist inpatient service, local care homes and daycare centres were asked to approach service users with Down syndrome. Assessments were conducted at a convenient place for participants; this was often at their home or their day centre.

### Participants

Men or women with a clinical diagnosis of Down syndrome, aged 45 or over who fulfilled the following inclusion criteria were included:

- Participants with stable and treated medical and/or mental health problems.
- Participants with sensory impairments that did not prevent them from being able to participate in the tasks.

Participants were required to understand simple verbal commands and attempt simple puzzles and games.

Exclusion criteria included the following:

- Known, unstable medical problems.
- Known, unstable psychosis or affective disorder.
- Clear history of previous Cerebrovascular Accident (CVA) or significant head injury.
- Participants with sensory impairments that prevented them from being able to participate in the tasks.

There was high medical and psychiatric co-morbidity in our study sample, which reflects the high levels of co-morbidity in this population. As this study was designed to be pragmatic, we had a low threshold for inclusion into the study, and considered referrer or carer report when considering suitability.

### Data collection

The principle researcher (ASi) met with participants and collected the data in one session if possible. Participants were offered a break half way through the assessment, or more as required. Occasionally, due to participant fatigue or other practical issues, data were collected over several sessions. At the end of the assessment, participants were given a choice of a gift voucher or small gift of a value of around £10.

Relatives or carers were asked to be present during the meeting and completed informant ratings while the participant completed the cognitive tests. Around half way through recruitment, the order of the tasks was counter-balanced, to reduce the potential effect of task position on performance.

### Pilot study

An assessment manual was developed by adapting the ACTB manual and selected clinical tabletop assessments. Tabletop assessments were selected by identifying tests that assessed cognitive abilities associated with similar brain regions as the ACTB (i.e. pre-frontal, hippocampal and cerebellar regions). Consideration was also given to the feasibility of the task.

Where appropriate, we amended the wording of some of the assessments from American English and to simpler English (for example, in CANTAB Intra-Extra Dimensional shift (IED), the instructions to participants was amended from identifying “correct” and “wrong” patterns, to identifying “right” and “wrong” patterns). The assessment was subsequently piloted on the first 3 participants. Following this, the CANTAB Motor Screening Task (MOT) task was added to the computer tasks as an initial teaching task.

### Primary outcome

The CANTAB Paired Associates Learning (PAL) was our primary outcome measure. It measures spatial associative memory. It has been shown to distinguish between those with Alzheimer’s disease and those without Alzheimer’s disease in the general population [25]. It has been used in research with children and adults with Down syndrome [21, 26, 27, 28, 29] and in clinical trials [30]. The outcome measures used in this study were first trial memory score (the number of patterns correctly located after the first trial) and stages completed (how many stages were successfully completed). For both measures, a higher score indicates a better result. This test is described further in S1 Box.

### Measures

S1 Box details the assessments used in this study.

#### ACTB

At the beginning of the cognitive assessment, we administered the CANTAB MOT, which is a CANTAB teaching test, designed to assess whether participants are able to use the touchscreen computer tablet. Participants were asked to press on flashing crosses on the touchscreen. Participants were given several attempts to do this task. If they were not able to attempt this task adequately, they were deemed to have failed the teaching stage for the computer tasks and did not attempt the rest of the computer-based tasks.

Subtests of the ACTB include CANTAB PAL (which measures spatial associative memory), CANTAB IED (measures set-shifting), Modified Dots Task (Cats and Frogs) (measures inhibitory control and working memory), CANTAB Simple Reaction Time (SRT) (originally designed as a measure of attention, but incorporated into the ACTB as a measure of cerebellar function), Finger Sequencing Task (Fingertapping) (measures motor sequencing) and NEPSY Visuomotor Precision (visuo-motor tracking and hand-eye coordination) [31].

The ACTB also contains a spatial memory task (Virtual Computer Generated Arena), which was not included in our battery due to inaccuracies in screen resolution, leading to poorly identifiable visual cues that were essential for validity of the task.

The Modified Dots Task and Finger Sequencing Task have been specifically developed for the ACTB. The Finger Sequencing Task can be conducted either using a computer mouse or a lever. Many participants were not familiar with operating a computer mouse, and this was therefore changed to a lever.

#### Tabletop tasks

For comparative purposes, we included several established tabletop tests. These were: NAID Object Memory [32], NAID Memory for Sentences [32], Tower of London Test [33], Verbal Fluency [34], Finger-Nose Test and Gait Assessment (Timed Get Up and Go Test) [35]. Object Memory, Memory for Sentences, Verbal fluency and the Tower of London test are widely used clinically in this population and have previously been used in studies with older people with Down syndrome [11, 36]. The Tower of London Test used was based upon a version developed for children [37] and adapted for use with people with intellectual disability. The Finger-Nose Test is a standardised version which has been used to assess motor coordination in older adults in the general population [38].

#### Informant ratings

The informant ratings analysed in this study were the Dementia questionnaire for people with Learning Disabilities (DLD)[39, 40], which includes a measure of short term memory and the Behaviour Rating Inventory for Executive Function (BRIEF—Parent Form) [41] for caregiver-reported symptoms of executive dysfunction and other frontal lobe features.

The BRIEF has been used in research with children and young adults with Down syndrome [21]. As the BRIEF parent version is designed for use with children, where appropriate, we adapted the wording to make it more applicable for an adult population.

The NEPSY Visuomotor Precision and BRIEF scores are designed to be transformed to percentiles using population norms for children. We therefore report raw scores only in our analyses.

For validity reasons, BRIEF questionnaires that had more than 14 questions not answered were not used in the analysis. Where no answers were provided for subsections, results are not reported.

#### Other assessments

The K-BIT II (Kaufman Brief Intelligence Test II) was used as an assessment of intelligence [42]. This generates separate verbal and non verbal raw subscales, which are combined to give a total raw score which is often used in analysis for individuals with intellectual disabilities as conversion of raw scores to population norms would result in a large proportion of participants scoring at floor.

Demographic and other relevant information, including severity of intellectual disability, medical history and pharmacological treatment was also collected. Severity of intellectual disability was recorded from informant report or case notes.

### Dementia diagnosis

Dementia diagnosis according to informant report was recorded at the time of assessment. Where applicable, the status of a dementia diagnosis at the time of assessment (or subsequently made by the treating clinician directly based on the findings of the assessment) was clarified from the clinical notes and/or by discussing this with the participant’s treating clinician. A consensus dementia diagnosis was then agreed by two members of the research team (ASi and ASt; both are specialist Intellectual Disability psychiatrists), taking into account the treating clinician’s dementia diagnosis as well as other relevant factors, for example, history of cognitive and functional decline and/or new onset of epilepsy. Clinical diagnosis of dementia in this population has been shown to be valid and reliable [43].

Participants were categorised into either a “dementia” or “no dementia group”. Those in the dementia group had a diagnosis of dementia or possible dementia. Some participants in the no dementia group had symptoms of cognitive concern, which may have been explained by other factors (such as physical or mental health problems) or were not enough to warrant a diagnosis of dementia. When reporting and discussing results, we will refer to the two groups as “dementia” and “no dementia” groups.

### Analysis

Numerical data were summarised using mean, standard deviation, median and range. Categorical data were summarised using count and percentages. Differences between the dementia and non-dementia groups were assessed using Chi-square test, Fisher's exact or Mann Whitney test as appropriate. For the cognitive tests, estimates of the difference and corresponding 95% confidence interval were presented. Correlation was used to assess concurrent validity.

As this was an exploratory study, p-values were not adjusted for multiple testing and therefore significant results should be interpreted with caution.

Analyses were undertaken in SPSS Version 21 [44] and Stata Version 12 [45].

### Participant details

A total of 50 participants were recruited. We conducted genetic analyses using a SNP array (Single Nucleotide Polymorphism array) on 39 individuals. Of these, 1 participant had disomy, 3 had mosaicism (including some with low level mosaicism) and rest had full trisomy. The participant with disomy was subsequently removed from analyses (see S1 Fig), as genetic testing did not show any evidence that the participant had Down syndrome. In total, five participants had mosaic Down syndrome, either documented in the clinical notes or confirmed with genetic testing; two participants with mosaicism had a diagnosis of dementia.

The mean age at first assessment was 52.7 years (*SD*: 6.06, range: 45.1–64.9 years), with mean age 55.6 years (*SD* 6.77) for those in the dementia group compared to 50.9 years (*SD*: 4.83) for those in the no dementia group. There was a significant difference in age between the two groups (*p* = 0.027).

## Results

S1 Fig summarises the flow of participants in the study.

### Demographic characteristics and medical conditions

Table 1 shows the demographic characteristics and common medical conditions of the study sample.

**Table 1:** Summary characteristics of the study sample (N = 49).

	Whole group/n (%)	Dementia/n (%)	No Dementia/n (%)	P value
**Gender**				
Male	23/49 (46.9%)	12/19 (63.2%)	11/30 (36.7%)	
Female	26/49 (53.1%)	7/19 (36.8%)	19/30 (63.3%)	0.070^a^
**Age**				
Below 50	20/49 (40.8%)	6/19 (31.6%)	14/30 (46.7%)	
50–54	14/49 (28.6%)	3/19 (15.8%)	11/30 (36.7%)	
55–59	5/49 (10.2%)	2/19 (10.5%)	3/30 (10.0%)	
60 and over	10/49 (20.4%)	8/19 (42.1%)	2/30 (6.7%)	0.027^b^
**Level of ID**				
Mild	13/35 (37.1%)	5/14 (35.7%)	8/21 (38.1%)	
Moderate/Severe	22/35 (62.9%)	9/14 (64.3%)	13/21 (61.9%)	0.886^a^
**Ethnic origin**				
White	41/48 (85.4%)	17/19 (89.5%)	24/29 (82.8%)	
African/Afro-Caribbean	3/48 (6.3%)	1/19 (5.3%)	2/29 (6.9%)	
Other	4/48 (8.3%)	1/19 (5.3%)	3/29 (10.3%)	0.839^c^
**Type of Accommodation**				
With family or friends	14/49 (28.6%)	6/19 (31.6%)	8/30 (26.7%)	
Adult placement	6/49 (12.2%)	1/19 (5.3%)	5/30 (16.7%)	
Sheltered/Supported/ Residential Care	29/49 (59.2%)	12/19 (63.2%)	17/30 (56.7%)	0.534^c^
**Medical conditions**				
Thyroid problems	20/48 (41.7%)	7/18 (38.9%)	13/30 (43.3%)	0.762^a^
Epilepsy	9/48 (18.8%)	5/19 (26.3%)	4/29 (13.8%)	0.451^c^
Falls	9/44 (20.5%)	4/16 (25.0%)	5/28 (17.9%)	0.702^c^
Congenital Cardiovascular problems	5/47 (10.6%)	2/18 (11.1%)	3/29 (10.3%)	1.000^c^
Hearing problems	8/46 (17.4%)	5/18 (27.8%)	3/28 (10.7%)	0.232^c^
Visual problems	23/48 (47.9%)	10/19 (52.6%)	13/29 (44.8%)	0.597^a^
Family history of dementia	7/33 (21.2%)	1/12 (8.3%)	6/21 (28.6%)	0.223^c^
Psychosis	7/45 (15.6%)	1/17 (5.9%)	6/28 (21.4%)	0.227^c^
Depression	15/45 (33.3%)	4/17 (23.5%)	11/28 (39.3%)	0.227^a^

^a^ Chi Squared value

^b^ Independent-Samples Mann-Whitney U Test

^c^ Fisher’s exact value

Five participants had documented hypercholesterolaemia and one had a history of hypertension. One participant had a diagnosis of diabetes (diet controlled Type II diabetes) and two were smokers. Thirteen participants had skin problems, including psoriasis and dermatitis. Five participants had abdominal problems, including abdominal pain and irritable bowel syndrome. Two participants were on anticoagulant treatment for deep vein thrombosis (DVT). One participant had a history of Bipolar Affective Disorder, and was receiving medication. Other mental health diagnoses recorded amongst participants included anxiety, OCD symptoms and behavioural problems.

### Use of the ATCB

In order to evaluate feasibility of the cognitive tests, we initially examined the proportion of participants who were able to attempt each of the ACTB and tabletop tasks (i.e. those who were able to start each of the tasks, after completing the teaching task, if appropriate). The number of participants that attempted the testing phase of the ACTB tasks ranged from 39 (81.3%) for the Modified dots task to 46 (93.9%) for the CANTAB SRT. The number of participants that attempted the testing phase of the tabletop tasks ranged from 34 (69.4%) for Object memory to 43 (87.8%) for Verbal fluency. Ten (52.6%) people with dementia were able to attempt all the ACTB tasks and ten (52.6%) were able to attempt all the tabletop tasks. 23 (76.7%) people in the no dementia group were able to attempt all the ACTB tasks and 21 (70.0%) attempted all the tabletop tasks. Reasons for participants not being able to attempt tasks included not passing the teaching stage, technical problems or the participant declining to attempt the task.

Table 2 shows distribution data for the specific cognitive assessments and differences between the dementia and no dementia groups. It also shows percentage of participants at floor and ceiling for each of the cognitive tasks. CANTAB IED stages completed and Modified Dots Task had particularly high percentages of participants at floor. See Box 1 for further details regarding calculation of floor and ceiling levels.

**Table 2:** Distribution data for cognitive measures and differences in cognitive tests between dementia and no dementia groups.

	Dementia (n = 19)					No Dementia (n = 30)						
	n	Mean	Median	Number at floor	Number at ceiling	n	Mean	Median	Number at floor	Number at ceiling	Median difference	P value ^c^
		(*SD*)	(Range)	(%)	(%)		(*SD*)	(Range)	(%)	(%)	(95% CI)	
**ACTB Tests:**												
**CANTAB PAL first trial memory score**	15	0.93	1	7	0	29	2.97	1	9	0	-1	0.099
		(1.16)	(0–4)	(46.7%)	(0.0%)		(3.77)	(0–15)	(31.0%)	(0.0%)	(-2 to 0)	
**CANTAB PAL stages completed**	15	1.40	2	4	0	29	3.21	2	2	2	-1	0.011
		(1.12)	(0–4)	(26.7%)	(0.0%)		(2.40)	(0–8)	(6.9%)	(6.9%)	(-2 to 0)	
**CANTAB IED stages completed**	15	0.60	0	9	0	29	2.41	1	14	0	0	0.159
		(0.83)	(0–2)	(60.0%)	(0.0%)		(3.11)	(0–8)	(48.3%)	(0.0%)	(-2 to 0)	
**IED errors block 1**	15	16.53	19	n/a	2	29	13.17	13	n/a	4	3	0.405
		(10.71)	(0–32)		(13.3%)		(11.49)	(0–29)		(13.8%)	(-3 to 10)	
**Modified Dots Task 2**^**nd**^ **stage**	11	0.36	0.33	10	0	21	0.46	0.33	14	4	0	0.876
		(0.16)	(0.00–0.58)	(90.9%)	(0.0%)		(0.35)	(0.08–1.00)	(66.7%)	(19.0%)	(-0.33 to 0.17)	
**Modified Dots Task 3**^**rd**^ **stage**	11	0.32	0.36	10	0	21	0.42	0.42	15	0	-0.09	0.155
		(0.14)	(0.12–0.52)	(90.9%)	(0.0%)		(0.19)	(0.03–0.91)	(71.6%)	(0.0%)	(-0.21 to 0.03)	
**CANTAB SRT median latency**	17	1556.5	1626	n/a	n/a	27	1186.8	1031	n/a	n/a	392	0.049
		(559.8)	(640–2350)				(616.5)	(351–2408)			(8.5 to 757)	
**Finger Sequencing task**	14	1.50	1	1	1	28	1.86	2	0	4	0	0.284
		(1.02)	(0–4)	(7.1%)	(7.1%)		(1.04)	(1–4)	(0.0%)	(14.3%)	(-1 to 0)	
**NEPSY Visuomotor Precision train and car**	10	8.50	10	0	0	25	11.08	11	0	0	-3	0.303
		(4.65)	(1–14)	(0.0%)	(0.0%)		(6.34)	(1–21)	(0.0%)	(0.0%)	(-7 to 1)	
**NEPSY Visuomotor Precision car and motorbike**	10	3.20	3	0	0	23	9.87	3	0	0	-3	0.013
		(1.99)	(1–8)	(0.0%)	(0.0%)		(8.46)	(2–28)	(0.0%)	(0.0%)	(-11 to -1)	
**Tabletop Tests:**												
**Verbal Fluency raw score**	14	2.93	3	4	n/a	29	6.3	6	2	n/a	-3	0.006
		(2.70)	(0–9)	(28.6%)			(4.10)	(0–17)	(6.9%)		(-5 to -1)	
**Verbal Fluency adjusted**	14	0.93	1	4	0	29	1.79	2	2	0	-1	0.002
		(0.73)	(0–2)	(28.6%)	(0.0%)		(0.86)	(0–4)	(6.9%)	(0.0%)	(-1 to 0)	
**Tower of London stages completed**	16	1.56	2	5	0	26	2.31	2	1	6	-1	0.102
		(1.26)	(0–3)	(31.3%)	(0.0%)		(1.19)	(0–4)	(3.8%)	(23.1%)	(-2 to 0)	
**Tower of London points**	11	3.27	3	7	0	25	3.88	3	7	2	0	0.710
		(2.49)	(0–7)	(43.8%)	(0.0%)		(3.32)	(0–10)	(26.9%)	(7.7%)	(-2 to 2)	
**Object Memory**	12	3.50	3.50	7	0	22	6.41	6.50	3	2	-3	0.007
		(2.94)	(0–9)	(43.8%)	(0.0%)		(2.40)	(1–10)	(12.0%)	(8.0%)	(-5 to -1)	
**Memory for Sentences**	14	9.71	11	3	0	28	14.64	10	1	0	-2	0.362
		(6.37)	(0–21)	(18.8%)	(0.0%)		(12.24)	(1–44)	(3.4%)	(0.0%)	(-9 to 3)	
**Finger-nose**	14	4.93	4.50	0	n/a	26	7.23	6.0	0	n/a	-2	0.130
		(2.92)	(1–10)	(0.0%)			(4.50)	(2–19)	(0.0%)		(-4 to 0)	
**Gait assessment**	12	15.76	14.55	3	n/a	26	14.08	11.96	0	n/a	2.35	0.155
		(4.26)	(7.82–23.17)	(20%)			(5.19)	(6.53–25.93)	(0.0%)		(-1.89 to 4.74)	
**Informant ratings:**												
**BRIEF**												
**Shift**	10	16.40	15.50			24	16.25	17.00			0	0.867
		(3.72)	(9–22)				(4.45)	(8–23)			(-3 to 2)	
**Inhibit**	11	16.55	17.00			24	15.75	15.00			1	0.636
		(3.73)	(11–22)				(4.10)	(10–24)			(-2 to 4)	
**Working memory**	11	22.00	20.00			24	20.33	21.00			1	0.587
		(4.86)	(15–30)				(5.21)	(10–30)			(-2 to 5)	
**BRI**	10	52.90	54.00			24	49.42	51.50			4	0.423
		(10.49)	(36–66)				(11.75)	(28–73)			(-6 to 12)	
**DLD**												
**Short Term Memory (STM)**	19	8.47	10			30	2.67	2.00			6	<0.001
		(4.01)	(0–13)				(3.03)	(0–11)			(4 to 9)	
**Sum Cognitive Scores**	19	26.47	26			30	12.43	12.50			15	<0.001
		(8.90)	(7–38)				(9.89)	(1–33)			(9 to 20)	
**Total Score**	19	46.53	43			30	25.10	25.50			23	<0.001
		(17.35)	(14–74)				(15.11)	(2–62)			(10 to 33)	
**K-BIT II:**												
**Total raw score**	19	9.74	6	0	0	30	23.17	14	0	0	-7	0.005
		(11.06)	(1–49)	(0.0%)	(0.0%)		(19.50)	(3–63)	(0.0%)	(0.0%)	(-22 to -2)	
**Verbal subscale raw score**	19	6.53	4	1	0	30	16.37	10	0	0	-6	0.002
		(7.61)	(0–34)	(5.3%)	(0.0%)		(13.33)	(1–47)	(0.0%)	(0.0%)	(-13 to -2)	
**Non verbal subscale raw score**	19	3.21	2	4	0	30	6.80	3	5	0	-1	0.184
		(4.16)	(0–15)	(21.1%)	(0.0%)		(6.92)	(0–20)	(16.7%)	(0.0%)	(-8 to 0)	

^c^ Independent samples Mann Whitney U test

**Box 1. Floor and Ceiling Levels**

For the majority of the tasks, floor was calculated as a score of zero (for: K-BIT II total raw score, K-BIT II verbal and non verbal subscales, CANTAB PAL stages completed and first trial memory score, CANTAB IED stages completed, Finger sequencing task, NEPSY visuomotor precision, Verbal fluency raw score and adjusted and Finger-nose test).

For NAID Object Memory, NAID Memory for Sentences, Tower of London points and stages completed, floor was calculated as a score of zero and/or did not pass teaching stage.

For Gait assessment, floor was calculated as being unable to mobilise without assistance.

In the Modified Dots Task, floor was calculated as 50% or under (as 50% of responses should be correct by chance alone). CANTAB SRT median latency is measured in units of time and, as it is on a continuum, floor and ceiling levels are not applicable.

Where applicable, ceiling levels were calculated as maximum possible score.

### Differences between dementia and no dementia groups

Of the ACTB tasks, significant differences between the dementia and no dementia groups were found for CANTAB SRT median latency, NEPSY Visuomotor Precision—Car and Motorbike and CANTAB PAL stages completed. However, 95% confidence intervals for the median difference did not include zero only for CANTAB SRT median latency and NEPSY Visuomotor Precision—Car and Motorbike. Of the other tasks, 95% confidence intervals did not include zero for K-BIT II total and verbal subscale raw scores, Verbal Fluency raw score, Object Memory, and DLD short term memory, sum of cognitive scores and total score, with the no dementia group performing better on these tests.

### Validity of the ACTB

In order to assess concurrent validity, we calculated correlation coefficients between measures that assessed the same or similar areas of cognition. That is, we compared prefrontal, hippocampal and cerebellar tasks from the ACTB with appropriate measures from the tabletop tests and informant ratings. The results are detailed below.

#### Validity—Prefrontal measures

Some of the CANTAB IED scores showed a significant mild to moderate correlation with Verbal fluency adjusted and Tower of London stages completed scores (from 0.40 to +/-0.45 depending on outcome measure used). The Modified dots task showed a significant moderate correlation with Verbal fluency adjusted and Tower of London stages completed (from 0.48 to 0.63 depending on outcome measure used). Very few of the BRIEF scales were significantly correlated with the CANTAB IED scores (only BRIEF working memory scores showed a mild to moderate correlation with some of the IED outcome measures, from -0.44 to 0.35) and none of the Modified dots scores were significantly correlated with the BRIEF scores.

#### Validity—Hippocampal measures

Both of the CANTAB PAL outcome measures were significantly correlated with Object memory (with both having a correlation of 0.35), and with DLD scores (from -0.45 to -0.56). There was no significant correlation between either of the CANTAB PAL measures and memory for sentences.

#### Validity—Cerebellar measures

There was a mild to moderate significant correlation between CANTAB SRT median latency and Finger-nose (-0.51) and Gait assessment (0.38) measures. There were also significant correlations between the NEPSY visuomotor precision outcome measures and the Finger-nose and Gait assessment measures (with the absolute magnitude of the correlation ranging from 0.43 to 0.51). The Finger sequencing task was significantly correlated with the Finger-nose task (0.42), but not Gait assessment.

## Discussion

We conducted a validation study of cognitive tests in older individuals with Down syndrome, with and without dementia. Although the majority of participants were able to attempt most of the tasks, some tasks had a large number of participants at floor. Furthermore, of the ACTB tasks, the only tests that were able to detect significant differences between the dementia and no dementia groups were the CANTAB Simple Reaction Time median latency, NEPSY Visuomotor Precision—Car and Motorbike and CANTAB Paired Associates Learning stages completed. In practical terms, the median difference and 95% confidence intervals show the magnitude of the difference in scores between the dementia and non dementia groups. When considering median difference, only CANTAB SRT and NEPSY Visuomotor precision—car and motorbike have 95% confidence intervals that do not include zero, and scores for a number of tests in the ACTB were similar when comparing dementia and no dementia groups. However, the DLD, for all three scales analysed, had p values of <0.001 and 95% confidence intervals that did not include zero, reflecting the large differences in DLD scores between the dementia and non-dementia groups.

### Strengths and limitations

This is the first study to fully assess the use of the ACTB in older adults with Down syndrome and compare its use in those with and without dementia. The study was designed to be pragmatic (i.e. conducted in real-life conditions as opposed to laboratory conditions) so that results could be directly applied to this population. It used manualised standardised assessments of cognitive skills in 49 adults with Down syndrome, a reasonable sample size when considering the difficulties of recruitment in this population.

Statistical analyses were hypothesis driven and limited to specific statistical tests that were relevant to the research question, thus reducing the possibility of multiple analyses and type I errors.

Regarding measurement of concurrent validity, we assessed the ACTB tests against tabletop tasks as well as informant ratings. The use of informant ratings provided a different measure of comparison that was less likely to be influenced by participant performance.

There are a number of limitations to this study, which mainly arise from the logistical issues of working with this population. There was a significant difference in age between the dementia and no dementia groups. This is to be expected, as it is well known that increasing age is associated with increasing risk of dementia in the general population [46], and in those with Down syndrome [27].

A larger proportion of the no dementia group were able to attempt the tasks as compared to the dementia group. Although breaks were given during the assessment and, on occasion, second and third assessments were arranged, the requirements to concentrate and pay attention to the tasks were sometimes difficult for participants. As the study aimed to focus on the ACTB, participants were encouraged to attempt the ACTB tasks, which may explain why a greater proportion attempted the ACTB tasks as opposed to the other tasks. The tasks were counterbalanced halfway through the assessment period, rather than alternating from the beginning of the study, which may have introduced some bias into the study.

We used clinical diagnoses to categorise participants into the “dementia” and “no dementia” groups. Some participants in the “no dementia” group may since have developed dementia.

Non-parametric methods were considered in the absence of suitable transformations of the data. These are less sensitive to extreme values, but are also potentially less robust, as they rely on ranking rather than the raw values of the data. Estimates (mean and median) and 95% confidence intervals of the median difference are presented in order to indicate the magnitude of the difference. As this was an exploratory study, and p values were not adjusted for multiple testing, results may need to be interpreted with caution.

### Floor Effects

The majority of data in this study did not fit a normal distribution, and much of the data was skewed. This is likely to be related to cognitive weaknesses in this group, resulting in large numbers of participants at floor for a number of outcome measures.

High floor levels have been a finding in other studies with similar populations. In a recent multinational study of adolescents and younger adults with Down syndrome, floor and ceiling effects were found in some of the cognitive tests, with some differences between the two groups [47]. In one large cross-sectional study of older adults with Down syndrome, the majority of participants with severe intellectual disability were unable to perform above floor on the majority of neuropsychological tests and were subsequently excluded from further analyses [11]. Interestingly, in a recent Spanish study of younger adults with Down syndrome, ceiling effects were found for PAL number of stages completed and SRT percent of correct answers [29].

### Differences between dementia and no dementia groups

In this study, of the 12 cognitive tests that were evaluated, only 5 were found to show a significant difference between the dementia and no dementia groups and only three of these tests were from the Arizona Cognitive Test Battery. Given that a diagnostic characteristic of dementia is deterioration in cognitive skills, we would have expected more cognitive differences to be detected between the dementia and no dementia groups.

One explanation for our findings may be that in this age range most individuals with Down syndrome are already showing significant cognitive deficits associated with Alzheimer’s disease despite not meeting the clinical threshold used for diagnosis. The similarities in the results of the cognitive tests may therefore reflect underlying similarities between the two groups.

As scores for a number of tests in the ACTB were similar when comparing dementia and no dementia groups, this indicates that some components of the ACTB may not, in its current form, be a useful cognitive battery in older individuals with Down syndrome but may need to be further adapted for use in this population.

### Comparison with cognitive function in children and younger adults with Down syndrome

Our sample had lower mean K-BIT II raw scores compared to that in the ACTB paper.

When comparing the CANTAB PAL first trial memory score results in our study to that in the original ACTB study, the mean was much lower in our study (2.27 compared to 7.42), which is likely to be a reflection of the differences in memory related to the difference in ages of people with Down syndrome in the two studies. Also, the percentage of participants at the floor of the CANTAB PAL first trial memory score was much higher in our study (36.4% versus 14.1%), which may explain why the distribution of results was not normally distributed. The means of the Modified Dots Task were also lower than the means of the same tests reported originally [21].

Furthermore, when comparing the means of the CANTAB SRT median latency and NEPSY Visuomotor Precision—train and car in our study to the original ACTB study, participants in our study showed poorer performance. This may highlight decline in fine motor skills with age in Down syndrome, though it is also possible that the original sample used for the development and validation of the ACTB was not typical of general population of individuals with Down syndrome.

Two recent cross sectional studies on cognition in adults with Down syndrome without dementia reflect our observation that in older adulthood, most individuals with Down syndrome may already show significant cognitive deficits associated with DAT, reporting a significant age-related decline in cognitive function and social adaptation skills [48] and lower neuropsychological function and adaptive skills in adults with Down syndrome over 40 years old, compared to those under 40 [49]. Language and short term memory, frontal lobe functions, visuospatial abilities and adaptive behaviour are particularly affected [49].

### Cognitive function in adults with Down syndrome and dementia

Significant cognitive differences using other test batteries have been found between older and younger adults with Down syndrome [14] and between individuals with Down syndrome with and without dementia [11]. These included tests of executive function and memory, including Tower of London, Verbal fluency, Object memory and Memory for sentences [11]. In our study, we also found a statistically significant difference comparing the dementia and no dementia groups on Object memory and Verbal fluency, but did not identify significant differences in the Tower of London or Memory for Sentences tasks.

The sample group in our study is older, and those in the no dementia group may already have experienced some of the changes in executive function and behaviour seen in the early stages of cognitive deterioration. However, we have also deliberately selected a sample that is representative of the older population with Down syndrome, and included individuals with a broader range of disability. This may explain why a number of participants, including those in the no dementia group, performed at floor for a number of the tests.

Although this study was not designed to measure attention in older people with Down syndrome and dementia, it became evident during the course of data collection that a number of participants were not able to attempt or complete the tasks due to limited attention, which is an important factor to consider in the design of future test batteries.

Das et al found that older adults with Down syndrome performed more poorly on cognitive tasks, particularly those that required planning and attention when compared to older adults with intellectual disability not due to Down syndrome and younger adults with intellectual disability both with and without Down syndrome [14]. Krinsky-McHale et al have suggested the addition of a selective attention task (a paper and pencil picture cancellation task) to a neuropsychological battery for dementia in Down syndrome and have found that changes in performance can be observed approximately 2 years before a clinical diagnosis of dementia [50].

Like the findings in Deb’s qualitative study [16], which documents general slowness in their study sample, in our study we also found that participants were generally slow on motor reaction time tasks, as reflected in the long median latency times seen in the CANTAB SRT.

In our study, there were significant differences between the dementia and no dementia groups in all three of the DLD outcome measures we used. In McCarron’s longitudinal study using objective and informant-based tests, the DLD was found to be most sensitive to tracking change in symptoms over time prior to a diagnosis of dementia [9].

### Recommendations for cognitive test battery for older adults with Down syndrome

We have made some suggestions regarding which of the tests in this battery are most useful when assessing cognitive skills in older people with Down syndrome. These are detailed in Table 3.

**Table 3:** Recommended cognitive tests to use in a cognitive test battery for older people with Down syndrome.

Test	Outcome measures	Comments
**CANTAB PAL**	- Stages completed	Used in many similar studies
**CANTAB SRT**	- Median latency	Difference in scores seen between dementia and no dementia groups.
**NEPSY Visuomotor precision**	- Train and Car	Difference in scores for Car and Motorbike seen between dementia and no dementia groups.
	- Car and Motorbike	
**Verbal fluency**	- Raw score	Difference in scores for raw score seen between dementia and no dementia groups. Easy to administer. Used in other studies.
**Tower of London**	- Stages succeeded on	Easy to administer. Used in many other studies.
**NAID Object memory**		Difference in scores seen between dementia and no dementia groups. Easy to administer. Used in other studies.
**DLD**	- Sum of cognitive scores	Difference in scores seen between dementia and no dementia groups. Easy to administer. Used in many other studies.
	- Total score	
**K-BIT II**	-Total raw score	Difference in scores seen between dementia and no dementia groups for total raw score and verbal subscale. Easy to administer. Used in other studies.
	- Verbal subscale	

In clinical practice, logistical issues such as access to hardware and software may limit the use of computer-based tests such as those in the CANTAB battery.

We suggest that cognitive test batteries to track change in older adults with Down syndrome need to include informant-rated tools.

## Conclusions

Some tests in the ACTB test battery (CANTAB PAL, CANTAB SRT, NEPSY Visuomotor Precision, Finger Sequencing) appear to be feasible and valid in older adults with Down syndrome, although for certain subtests (including CANTAB IED, Modified Dots Task, Finger Sequencing, NEPSY Visuomotor precision—Train and Car and CANTAB PAL first trial memory score), scores may be similar when comparing those with and without dementia. This may be explained by a minimal difference in specific cognitive skills between those with a diagnosis of dementia and those without.

Several of the tests used in this study may be helpful in assessing cognitive skills and cognitive decline in older people with Down syndrome and may be useful in preventative treatment trials (these include CANTAB PAL, CANTAB SRT, NEPSY Visuomotor Precision, Verbal Fluency, Tower of London, NAID Object Memory, DLD, K-BIT II). However, the ACTB as a whole is unlikely to show change in cognitive skills in those who have a diagnosis of dementia and it may be necessary to also use informant-rated assessment tools in the Down syndrome population with dementia. In addition it may be helpful to consider including an assessment of attention in any cognitive battery used with older adults with Down syndrome.

Nevertheless, repeated assessments over time of specific cognitive skills using subtests of the ACTB will be useful in future research including epidemiological studies and clinical trials.

## Supporting Information

S1 Box Summary of Cognitive Assessments. (DOC)

S1 Fig Flow Diagram. (DOC)
