# Supplementary material for: Assessing Specific Cognitive Deficits Associated with Dementia in Older Adults with Down Syndrome: Use and Validity of the Arizona Cognitive Test Battery (ACTB)
Source: PLoS One. 2016 May 12;11(5):e0153917. doi: 10.1371/journal.pone.0153917 (PMC4865091; doi:10.1371/journal.pone.0153917)
Supplement: S1 Box — (DOC) [file pone.0153917.s001.doc]

# **S1 Box – Summary of Cognitive Assessments**

| **Test** | Description | Outcome measure/s used (range) | Cognitive skill measured |
| --- | --- | --- | --- |
| **ACTB Tests** |  |  |  |
| **Hippocampal tests:** |  |  |  |
| CANTAB Paired Associates Learning (PAL) | Six white squares open up in turn, revealing patterns behind some of them. Participants need to identify which pattern was behind which square. Stages range from 1 pattern in 1 of 6 squares, to 8 squares each with 8 different patterns. There are 8 stages in total. | First trial memory score (0-26)  Stages completed (0-8) | Spatial associative memory |
| **Prefrontal tests:** |  |  |  |
| CANTAB Intra-Extra Dimensional shift (IED) | Participants are shown two patterns and are asked to work out the rule and press the box with the “right” pattern in it. The rule changes and becomes increasingly complex as the test progresses. | Stages completed (0-9) Errors block 1 | Set-shifting |
| Modified dots task (Cats and Frogs) | Participants are initially taught a rule for which button to press when a picture of a cat comes on the screen. In the second stage, they are taught a new rule for when a frog comes on the screen. In the third stage, participants are required to shift between the cat and frog rules accordingly. | Percentage correct 2^nd^ stage (0-100%)  Percentage correct 3^rd^ stage (0-100%) | Inhibitory control Working memory |
| **Cerebellar tests:** |  |  |  |
| CANTAB Simple Reaction Time (SRT) | Participants are asked to press the button on the press pad as soon as they see a white square appear in the centre of a black computer screen. | Median latency score | Cerebellar function  Attention |
| Finger sequencing task (Fingertapping) | Measures finger sequencing generated by tapping a computer mouse or lever with a number of fingers (from 1 to 4) in succession. Repeated on both right and left hands. | Maximum sequences reached (0-4) | Motor sequencing |
| NEPSY Visuomotor precision | Participants draw a line around a track, without going outside the track lines and without turning the paper. There are three tracks to complete. The width, length and complexity of the track increases with each track. Higher scores indicate increased ability. | First and second tracks – train and car raw scores (30-0)  Second and third tracks – car and motorbike raw scores (52-0) | Visuo-motor tracking  Hand-eye coordination |
| **Table top tests** |  |  |  |
| **Hippocampal tests:** |  |  |  |
| NAID Object memory | Participants are shown ten everyday items (a comb, a key, a letter, a 10 pence coin, a spoon, a watch, a notepad, a purse, a pencil and a toothbrush). Any items participants are not able to name are not used in testing. In the testing stage, two items are displayed and participants are asked to name them. Whilst the participant is looking away, one item is covered. Participants are asked to name which item is covered. This is repeated with another two items. This is then repeated with 3, 4, 5 and 6 items. | Number of objects remembered (0-10) | Object memory |
| NAID Memory for sentences | During the teaching phase, the assessor asks the participant to repeat the words “watch” and “lamp” back to them. During the testing phase, participants are asked to repeat back a sentence. The sentences become progressively longer. | Number of words remembered (0-48) | Verbal (immediate) memory |
| **Prefrontal tests:** |  |  |  |
| Tower of London | The researcher and participant both have a board with a green, a red and a blue ball in a specific starting position. In the teaching phase, participants are shown a 1 move configuration and are asked to make their board look like the one the researcher has. In the test phase, participants have to make increasingly complex configurations with as few moves as possible, moving one ball at a time. In our study, we used two 2- and 3-move configurations and one 4-move configuration. | Stages completed (0-4)  Points (0-10) | Executive functioning (planning) |
| Verbal Fluency | Participants are asked to name as many animals they can think of in one minute. The total number of animals is adjusted using a scale, where 1-5 animals = 1, 6-10 animals = 2, 11-15 animals = 3 and 16 and over = 4. | Raw score (0- )  Adjusted score (0-4) | Verbal fluency |
| **Cerebellar tests:** |  |  |  |
| Finger-nose test | Participants are asked to touch their nose and then touch a red spot of 2cm diameter which is 45 cm away from them. | Number of times achieved in 20 seconds (0- ) | Cerebellar function (motor co-ordination) |
| Gait assessment - timed up and go test | Participants are timed to see how long it takes for them to get up from a chair, walk a distance of 3 meters, turn around, walk back to the chair and sit back down. Participants have one practice and then two test trials. | Average of the two testing trials. If only a practice trial was completed, this was included in the analysis for completeness. | Functional mobility |
| **Informant ratings** |  |  |  |
| Dementia questionnaire for people with Learning Disabilities (DLD) | Carers are asked to rate various items relating to the cognitive and social function of the individual with intellectual disability over the past two months. Sums of cognitive scores (SCS) and sums of social scores (SSS) are calculated, which combine to form a total score. Higher scores indicate a higher deficit. | Short Term memory subscale (0-14)  Sum of cognitive scores (SCS) (0-44)  Total score (0-104) | Short term memory Long term memory Orientation |
| Behaviour Rating Inventory for Executive Function (BRIEF – Parent Form) | This is an 86 item questionnaire for use for people between 5-18 years. Carers rate behaviours in the past 6 months according to whether the behaviour has occurred Never, Sometimes or Often. Results are then calculated to generate 8 subscale scores. | Shift subscale (8-24)  Inhibit subscale (10-30)  Working Memory subscale (10-30)  Behavioural Regulation Index (made up of Inhibit, Shift and Emotional Control subscales) (28-84) | Executive functioning |
| **Other assessments** |  |  |  |
| K-BIT II | The K BIT II (Kaufman Brief Intelligence Test) is a measure of verbal and non-verbal intelligence and is appropriate for use for people between 4 and 90 years of age. | Total raw score (0-108)  Verbal subscale raw score (0-46)  Non verbal subscale raw score (0-154) | Verbal intelligence  Non verbal intelligence |
