# Supplementary material for: Assessing Specific Cognitive Deficits Associated with Dementia in Older Adults with Down Syndrome: Use and Validity of the Arizona Cognitive Test Battery (ACTB)
Source: PLoS One. 2016 May 12;11(5):e0153917. doi: 10.1371/journal.pone.0153917 (PMC4865091; doi:10.1371/journal.pone.0153917)
Supplement: S1 Fig — (DOC) [file pone.0153917.s002.doc]

# **S1 Figure – Flow Diagram**

Potential participants approached by intellectual disability teams and day centres

75 people contacted by researcher

Reasons not entered into study:

Unable to attempt cognitive tests (7)

Did not want to take part (5)

Unable to arrange assessment before recruitment end date (4)

Significant sensory impairment (2)

Below age limit (2)

Unclear diagnosis of Down syndrome (2)

Deceased (2)

Behavioural problems (1)

50 participants assessed

19 with dementia or possible dementia

30 with no dementia

1 participant excluded as genetic testing did not show any evidence of Down syndrome
